# Diagnostic accuracy, clinical characteristics, and prognostic differences of patients with acute myocarditis according to inclusion criteria

**DOI:** 10.1093/ehjqcco/qcad061

**Published:** 2023-10-31

**Authors:** Roman Roy, Antonio Cannata, Mohammad Al-Agil, Emma Ferone, Antonio Jordan, Brian To-Dang, Matthew Sadler, Aamir Shamsi, Mohammad Albarjas, Susan Piper, Mauro Giacca, Ajay M Shah, Theresa McDonagh, Daniel I Bromage, Paul A Scott

**Affiliations:** King's College London British Heart Foundation Centre of Excellence, School of Cardiovascular and Metabolic Medicine & Sciences, London SE5 9NU, UK; King's College Hospital NHS Foundation Trust, London SE5 9RS, UK; King's College London British Heart Foundation Centre of Excellence, School of Cardiovascular and Metabolic Medicine & Sciences, London SE5 9NU, UK; King's College Hospital NHS Foundation Trust, London SE5 9RS, UK; King's College Hospital NHS Foundation Trust, London SE5 9RS, UK; King's College London British Heart Foundation Centre of Excellence, School of Cardiovascular and Metabolic Medicine & Sciences, London SE5 9NU, UK; King's College Hospital NHS Foundation Trust, London SE5 9RS, UK; King's College Hospital NHS Foundation Trust, London SE5 9RS, UK; King's College London British Heart Foundation Centre of Excellence, School of Cardiovascular and Metabolic Medicine & Sciences, London SE5 9NU, UK; King's College Hospital NHS Foundation Trust, London SE5 9RS, UK; King's College Hospital NHS Foundation Trust, London SE5 9RS, UK; Princess Royal University Hospital, Orpington, London BR6 8ND, UK; King's College Hospital NHS Foundation Trust, London SE5 9RS, UK; King's College London British Heart Foundation Centre of Excellence, School of Cardiovascular and Metabolic Medicine & Sciences, London SE5 9NU, UK; King's College London British Heart Foundation Centre of Excellence, School of Cardiovascular and Metabolic Medicine & Sciences, London SE5 9NU, UK; King's College Hospital NHS Foundation Trust, London SE5 9RS, UK; King's College London British Heart Foundation Centre of Excellence, School of Cardiovascular and Metabolic Medicine & Sciences, London SE5 9NU, UK; King's College Hospital NHS Foundation Trust, London SE5 9RS, UK; King's College Hospital NHS Foundation Trust, London SE5 9RS, UK

**Keywords:** Myocarditis, Diagnosis, Cardiac magnetic resonance imaging, Endomyocardial biopsy, ICD codes

## Abstract

**Introduction:**

The diagnosis of acute myocarditis (AM) is complex due to its heterogeneity and typically is defined by either Electronic Healthcare Records (EHRs) or advanced imaging and endomyocardial biopsy, but there is no consensus. We aimed to investigate the diagnostic accuracy of these approaches for AM.

**Methods:**

Data on ICD 10th Revision(ICD-10) codes corresponding to AM were collected from two hospitals and compared to cardiac magnetic resonance (CMR)-confirmed or clinically suspected (CS)-AM cases with respect to diagnostic accuracy, clinical characteristics, and all-cause mortality. Next, we performed a review of published AM studies according to inclusion criteria.

**Results:**

We identified 291 unique admissions with ICD-10 codes corresponding to AM in the first three diagnostic positions. The positive predictive value of ICD-10 codes for CMR-confirmed or CS-AM was 36%, and patients with CMR-confirmed or CS-AM had a lower all-cause mortality than those with a refuted diagnosis (*P *= 0.019). Using an unstructured approach, patients with CMR-confirmed and CS-AM had similar demographics, comorbidity profiles and survival over a median follow-up of 52 months (*P *= 0.72). Our review of the literature confirmed our findings. Outcomes for patients included in studies using CMR-confirmed criteria were favourable compared to studies with endomyocardial biopsy-confirmed AM cases.

**Conclusion:**

ICD-10 codes have poor accuracy in identification of AM cases and should be used with caution in clinical research. There are important differences in management and outcomes of patients according to the selection criteria used to diagnose AM. Potential selection biases must be considered when interpreting AM cohorts and requires standardization of inclusion criteria for AM studies.

## Graphical Abstract

**Graphical Abstract:**
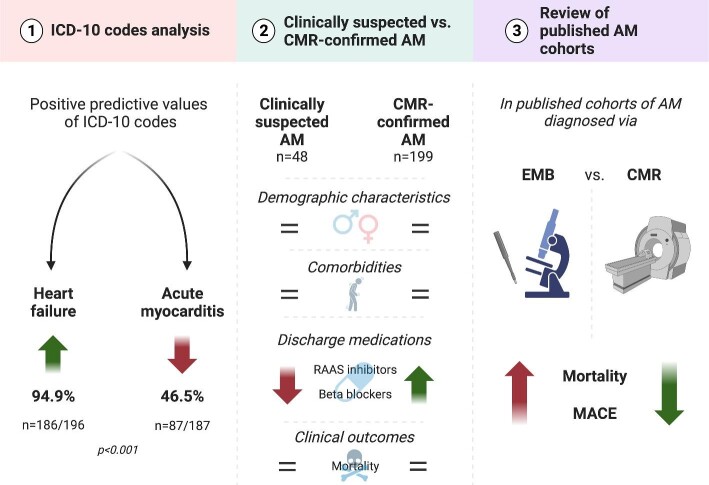
Created with BioRender.com

Key learning points **What is already known:**

- Acute myocarditis (AM) is a heterogeneous disease with varied clinical presentations, many clinical mimics, and limitations of diagnostic tests.
- AM is typically diagnosed either clinically, or by cardiac magnetic resonance imaging, or endomyocardial biopsy.

**What this study adds:**

- ICD-10 codes, often used in AM research, lack sensitivity and specificity in case identification.
- There are important differences in management and outcomes of patients depending on specific criteria used to diagnose AM.
- Potential selection biases stemming from diagnostic criteria must be borne in mind when interpreting cohorts of AM patients.

## Introduction

Acute myocarditis (AM) is an inflammatory disease of the myocardium associated with heterogeneous clinical presentations.^1,2^ These include acute coronary syndrome-like symptoms, heart failure (HF) with or without cardiogenic shock, and arrhythmias/aborted sudden cardiac death. The investigation of suspected AM is also complex. Despite consensus that the gold standard diagnostic test for AM is endomyocardial biopsy (EMB)^1,3^ and evidence that EMB is a safe albeit invasive test,^4–6^ routine use in clinical practice remains limited. Cardiac magnetic resonance (CMR) imaging has emerged at the forefront of diagnostic criteria proposed by the European Society of Cardiology (ESC) Working Group position statement^1^ and the International Consensus Group on CMR Diagnosis of Myocarditis.^7,8^ Cardiac magnetic resonance provides information not only on aetiology of heart disease but also on cardiac function, which may have value in risk stratifying patients with AM.^9^ However, although it is non-invasive, it requires resources and user expertise, which may limit use in routine clinical practice.

This diversity in clinical presentation and limited acceptability/availability of the key diagnostic tests can lead to a significant diagnostic challenge in AM. Consequently, patients are frequently diagnosed with clinically suspected AM (CS-AM). This is defined by the ESC as patients who do not have evidence of AM through EMB or CMR but exhibit at least one clinical presentation and meet at least one diagnostic criterion. In addition, they should not have coronary artery disease (CAD) on angiography or any pre-existing cardiovascular diseases or extra cardiac diseases that could explain the presentation.^1^ However, it is unclear if CS-AM patients have comparable presentations, treatments, and outcomes to those confirmed through CMR or EMB.

Despite its complexity, accurate diagnosis of AM is important for several reasons. First, AM can mimic other cardiovascular conditions, such as acute coronary syndromes, whose treatment is very different from that of AM. Second, accurate diagnosis is essential when developing and interpreting the evidence base, which informs guidelines. Accurate case identification will affect the quality of the evidence base, the external validity of studies and whether the studied populations truly represent AM. The American College of Cardiology/American Heart Association recently acknowledged the importance of accurate diagnosis in AM in their 2022 clinical practice guidelines for HF, labelling the definition, detection, and management of myocarditis an important evidence gap and future research direction.^10^ Furthermore, many clinical studies outside of AM use International Classification of Diseases 10th Revision (ICD-10) diagnostic codes to identify cohorts. Given the diagnostic challenges of AM it is unclear how well ICD-10 coding performs in this setting.

The aims of this study were therefore to:

1. Describe the complexity of accurately diagnosing AM.
2. Assess the impact of different available diagnostic criteria on reported outcomes in AM.

## Methods

### Ethics and study overview

This project operated under London South East Research Ethics Committee approval (18/LO/2048) granted to the King's Electronic Records Research Interface.

We performed three complementary analyses to investigate the complexity and importance of accurate diagnosis of AM:

1. We performed a retrospective validation of patients discharged with AM-related diagnostic ICD-10 codes at our Centre, using HF ICD-10 codes as a control.
2. We described the differences in CMR-confirmed AM compared to CS-AM using data from our Centre.
3. We reviewed the literature for studies using ICD-10 codes, CS-AM, CMR- and EMB-confirmation in their inclusion criteria and investigated the association with reported outcomes.

### Validation of ICD-10 diagnostic codes in AM

#### ICD-10 codes for acute myocarditis case identification

We performed a retrospective analysis of all unique admissions with AM-related ICD-10 codes at two hospitals within our hospital Trust (King's College Hospital, London, UK and Princess Royal University Hospital, Orpington, UK) between 2008 and 2020. Only patients aged 18 years and above were included. ICD-10 codes used are summarized in *Table 1*, with patients included if the relevant ICD-10 code was in one of the first three diagnostic coding positions. International Classification of Diseases 10th Revision codes were selected based on the literature^11^ as well as consensus amongst the study authors. We used the first three coding positions to maximize the amount of AM cases identified. Patients with suspected COVID-19- or vaccination-associated AM, as well as patients with sarcoidosis were excluded from this study.

**Table 1:** ICD-10 codes corresponding to a diagnosis of acute myocarditis and heart failure

Acute myocarditis		Heart failure	
B33.2	Viral carditis	I11.0	Hypertensive heart disease with (congestive) heart failure
I01.2	Acute rheumatic myocarditis	I25.5	Ischaemic cardiomyopathy
I09.0	Rheumatic myocarditis	I42.0	Dilated cardiomyopathy
I40	Acute myocarditis	I42.9	Cardiomyopathy, unspecified
I41	Myocarditis in diseases classified elsewhere	I50.0	Congestive heart failure
I51.4	Myocarditis, unspecified	I50.1	Left ventricular failure
		I 50.9	Heart failure, unspecified

Discharge summaries were screened, and patients who did not have an admission related to AM were excluded (e.g. a past medical history of AM or elective/unrelated admissions). After initial screening of the discharge summary, various data points were collected from the electronic health records (EHR), including clinical presentation, cardiac biomarkers, 12-lead electrocardiogram, echocardiogram, CMR findings, and clinical outcomes. Admissions were excluded if there was insufficient evidence of AM (e.g. lack of required investigations such as cardiac biomarkers or echocardiogram), CAD had not been excluded, or there was a clear alternative diagnosis apparent. Admissions were categorized as confirmed AM if proven by either CMR or EMB, or CS-AM in the absence of CMR or EMB confirmation but if ESC Position Statement/AHA Scientific Statement criteria were met.^1,12^ The 2018 Lake Louise criteria for AM^8^ were used as the CMR criteria for diagnosis. We then compared all-cause mortality between patients with confirmed vs. refuted AM.

#### Comparison with HF ICD-10 codes

To standardize results for coding practices at our Centre, we used the same methodology to validate admissions for patients with HF as a comparator, using ICD-10 codes with well-described sensitivity and specificity.^13^ Using relevant ICD-10 codes (*Table 1*), we identified unique admissions at our Centre with suspected HF. Heart failure cases were identified using the National Institute for Cardiovascular Outcomes Research data, which identifies cases based on ICD-10 codes in the first diagnostic position. Therefore, to allow an analogous comparison between HF and AM, we used only patients with relevant ICD-10 codes in the first coding position for both presentations for this portion of the analysis. Given the larger number of HF admissions, HF cases were date-matched to AM cases, with all HF cases being within one day of a matched AM presentation. Some days had no corresponding HF admissions while others had multiple corresponding HF admissions. All relevant HF admissions were used for this analysis. A diagnosis of HF was confirmed or refuted on detailed review of clinical presentation, and laboratory and imaging findings from the EHR by a cardiologist according to ESC clinical practice guidelines.^14^

### Comparison of clinically suspected and CMR-confirmed AM

We investigated differences in characteristics, management, and outcomes of patients with CS-AM compared to CMR-confirmed AM. We included all consecutive patients admitted with AM at our hospital Trust, as described elsewhere.^2^ Briefly, patients ≥18 years were identified using an open-source retrieval system for unstructured clinical data (CogStack^15^) using the keywords ‘myocarditis’ or ‘myopericarditis’ in hospital/ITU discharge summaries or death notifications. Further patients were also identified using relevant ICD-10 codes (*Table 1*). Discharge summaries for all unique admissions were screened, and data on clinical presentations, laboratory/imaging findings, treatment, and outcomes were collected from the EHR in those with possible AM. Patients meeting ESC Criteria^1^ but without CMR or EMB confirmation were classified as CS-AM. Patients meeting Lake Louise CMR criteria^8^ or undergoing confirmatory EMB were classified as CMR/EMB confirmed AM. The primary outcome for this analysis was all-cause mortality.

### Differences between published AM cohorts with varying diagnostic criteria

We assessed the effect of different inclusion criteria on outcomes in studies of AM. We identified studies by a non-systematic literature review, based on expertise of the authors and reviewing reference lists of published articles. We collected data pertaining to inclusion and exclusion criteria as well as clinical outcomes. We then classified studies into those including patients with ICD-10 code-identified AM, CS-AM, primarily CMR-confirmed AM, and EMB-confirmed AM. We categorized studies as primarily CMR-confirmed AM if >50% of patients underwent CMR diagnostic for AM (detailed in *Table 3*). We performed a qualitative synthesis of the extracted data.

### Statistical analysis

Data are presented as mean and standard deviation, median and interquartile range or number and percentage as appropriate. Comparison of means was performed using the analysis of variance (ANOVA) test for normally distributed continuous variables, and the Mann–Whitney test for non-normally distributed variables. Comparison of proportions was performed using the Chi Square test. All statistical tests were two-sided. A *P* value < 0.05 was considered statistically significant. All statistical analysis was performed using IBP SPSS Statistics version 28 and R.

## Results

### Validation of ICD-10 diagnostic codes in AM

#### ICD-10 codes for acute myocarditis case identification

**Figure 1:**
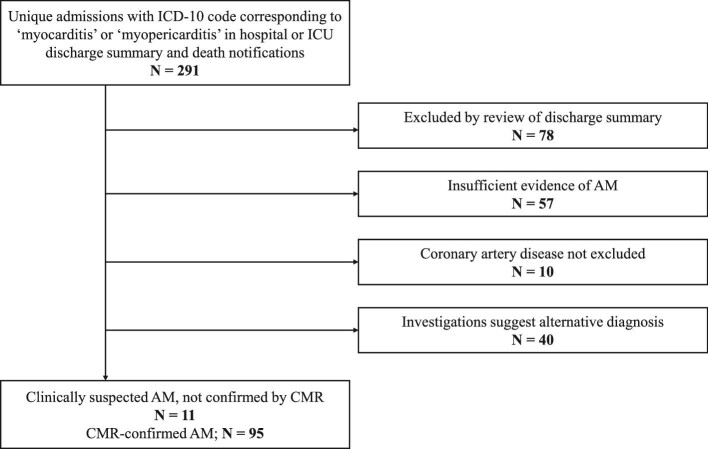
Study flowchart for validation of ICD-10 codes corresponding to acute myocarditis. ICD-10 codes corresponding to a diagnosis of AM in the first three coding positions were used. AM: acute myocarditis; CMR: cardiac magnetic resonance imaging; ICD-10: International Classification of Diseases 10th Revision; and ICU: intensive care unit.

A total of 291 unique admissions between 2008 and 2020 with ICD-10 codes corresponding to AM in the first three coding positions were identified (*Figure 1*). Of these, 26.8% (*n* = 78) could be excluded from a diagnosis of AM on review of the discharge summary. Following this, the full EHRs were reviewed. 19.6% (*n* = 57) admissions had insufficient evidence of AM, 3.4% (*n* = 10) had not had CAD excluded as a culprit for the presentation, and 13.7% (*n* = 40) had an apparent alternative diagnosis. This left 95 cases of CMR-proven AM, and 11 cases of CS-AM (without CMR confirmation). Therefore, the positive predictive value (PPV) of ICD-10 codes for CS or CMR-confirmed AM was 36.4%, and 32.6% for purely CMR-confirmed AM.

**Figure 2:**
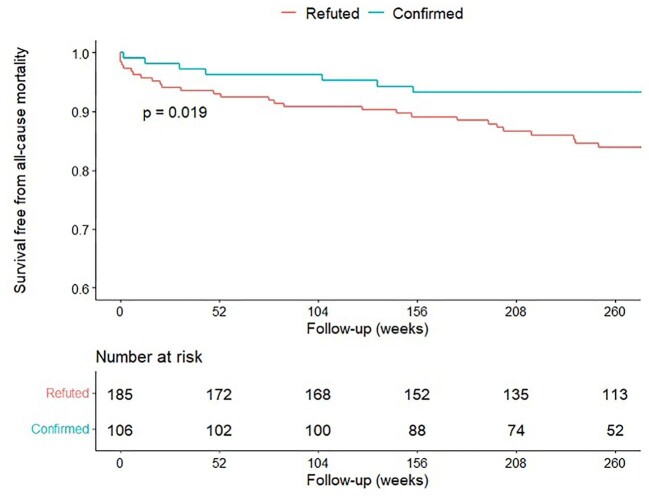
Kaplan–Meier curve for all-cause mortality in patients with relevant ICD-10 codes with refuted vs. confirmed AM.

A comparison of patients with relevant ICD-10 codes who, after review of the EHRs had MRI-proven or CS-AM vs. refuted AM (*n*=106 vs. *n* = 185) showed that patients with refuted AM had significantly higher all-cause mortality over a mean 67 (IQR 45–100) months follow-up (*P *= 0.019; *Figure 2*).

#### Comparison with HF ICD-10 codes

**Figure 3:**
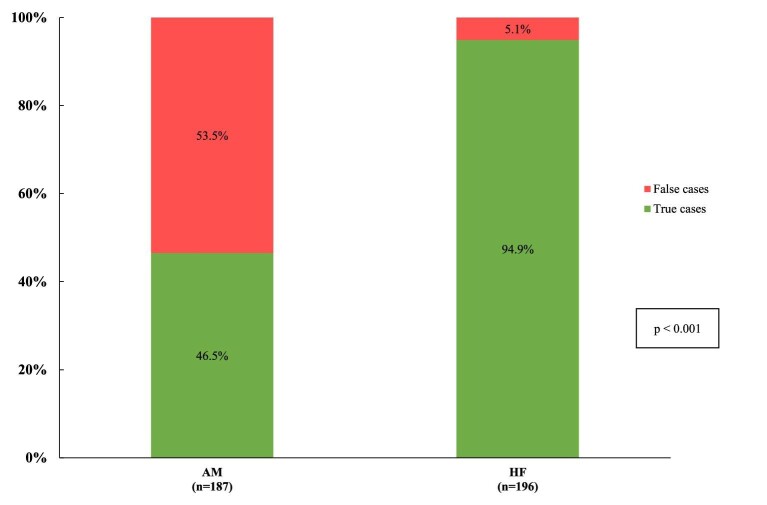
Analysis of validity of ICD-10 codes corresponding to clinically suspected acute myocarditis (CS-AM) or cardiac magnetic resonance (CMR)-confirmed acute myocarditis and heart failure (HF) diagnosis.

A total of 187 unique admissions with ICD-10 codes corresponding to AM in the first coding position were identified between 2008 and 2020. Of these only 46.5% (*n* = 87) met criteria for CS-AM or CMR-confirmed AM. In comparison, a total of 196 unique HF admissions were identified, of which 94.9% (*n* = 186) were true HF cases on review of the EHRs. The PPV of relevant ICD-10 codes was significantly lower for AM than it was for HF (*P *< 0.001; *Figure 3*).

### Comparison of clinically suspected and CMR-confirmed AM

**Figure 4:**
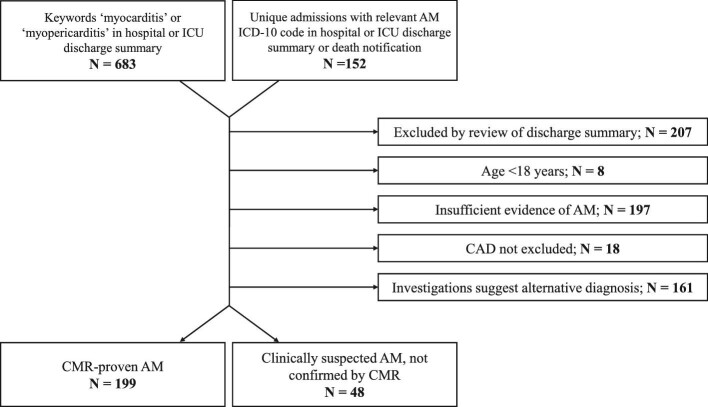
Study flowchart for comparison of clinically suspected AM vs. CMR-confirmed AM patients. ICD-10 codes corresponding to a diagnosis of AM in the first three coding positions were used. AM: acute myocarditis; CAD: coronary artery disease; CMR: cardiac magnetic resonance imaging; ICD-10: International Classification of Diseases 10th Revision; and ICU: intensive care unit.

Screening hospital and ICU discharge summaries for keywords ‘myocarditis’ or ‘myopericarditis’ revealed 683 unique patients (*Figure 4*). Screening death notifications identified three additional patients. A further 152 unique patients were identified by searching for admissions with ICD-10 codes corresponding to myocarditis in the first three coding positions. Discharge summaries were screened for eligibility and 207 cases were excluded due to not being a discharge diagnosis or death notification of AM. A further eight patients were excluded as they were younger than 18 years old at time of admission. Next, the full EHRs were evaluated and data on diagnostic criteria extracted. From this we excluded 197 patients with insufficient evidence of AM, 18 in whom CAD was not sufficiently excluded and 161 in whom an alternative diagnosis was evident. Therefore, a total of 48 patients had CS-AM that was not proven by CMR or EMB, and 199 patients had CMR-confirmed AM, with one of these patients also undergoing EMB.

#### Baseline characteristics, clinical presentation, and management

Baseline characteristics for the CMR-confirmed and CS-AM cohorts are shown in *Table 2*. Overall, 66% (*n* = 163) of patients were male with a mean age of 40.0 ± 16.3 years. There were no significant differences in age, sex, or ethnicity between confirmed vs. suspected AM patients. Clinical presentation of patients was similar, although there was a trend toward more arrhythmia presentations and less breathlessness amongst the CMR-confirmed AM population compared to the CS-AM population (*P *= 0.081). There were no differences in comorbidities, with patients most frequently having premorbid hypertension (15%; *n* = 37/247) and autoimmune disorders (11%; *n* = 26/247), and only rarely previous myocarditis (3.3%; *n* = 8/247). Baseline medications were similar, although the CS-AM cohort was more likely to be taking a renin-angiotensin-aldosterone-system (RAAS) inhibitor than the CMR-confirmed group (19.0% vs. 7.3%, *P *= 0.024). Amongst patients with CS-AM, only 14.6% (*n* = 7) underwent CMR. This was performed a mean of 63 (IQR 2–119) days after admission, with four patients undergoing CMR within 10 days and three significantly later.

**Table 2:** Baseline characteristics, admission investigation, in-hospital management, and clinical outcomes of patients with clinically suspected vs. cardiac magnetic resonance-confirmed acute myocarditis

Variable		Overall *N* = 247	Clinically suspected *N* = 48	CMR-confirmed *N* = 199	Significance *P*-value
**Baseline characteristics**					
Male sex *N (%)*		163 (66%)	33 (69%)	130 (65%)	0.653
Age at presentation *(years)*		40.0 ± 16.3	43.3 ± 16.0	39.2 ± 16.3	0.087
Race *N (%)*	Caucasian	123 (51%)	24 (51%)	99 (52%)	0.886
	Black	78 (33%)	17 (36%)	61 (32%)	
	Asian	10 (4.2%)	2 (4.3%)	8 (4.2%)	
	Other	28 (12%)	4 (8.5%)	24 (12%)	
Clinical presentation *N (%)*	Chest pain	183 (74%)	34 (71%)	149 (75%)	0.081
	Breathlessness	45 (18%)	13 (27%)	32 (16%)	
	Arrhythmia	19 (7.7%)	1 (2.1%)	18 (9.0%)	
Comorbidities *N (%)*	Autoimmune disorders	26 (11%)	2 (4.2%)	24 (12%)	0.106
	Previous myocarditis	8 (3.3%)	1 (2.1%)	7 (3.6%)	>0.999
	Hypertension	37 (15%)	11 (23%)	26 (13%)	0.092
	Dyslipidaemia	14 (5.7%)	3 (6.2%)	11 (5.6%)	0.741
	Diabetes	16 (6.5%)	4 (8.3%)	12 (6.1%)	0.526
	CKD	9 (3.7%)	1 (2.1%)	8 (4.1%)	>0.999
	PAD	1 (0.4%)	0 (0%)	1 (0.5%)	>0.999
	Previous MI	2 (0.8%)	1 (2.1%)	1 (0.5%)	0.354
	Alcohol excess	5 (2.0%)	1 (2.1%)	4 (2.0%)	>0.999
Baseline medications *N (%)*	RAAS inhibitor	23 (9.6%)	9 (19%)	14 (7.3%)	**0.024**
	Beta blocker	17 (7.1%)	2 (4.3%)	15 (7.8%)	0.537
	MRA	0 (0%)	0 (0%)	0 (0%)	
	Diuretic	7 (2.9%)	2 (4.3%)	5 (2.6%)	0.626
	Immunosuppressant	21 (8.8%)	2 (4.3%)	19 (9.9%)	0.386
	Statin	23 (9.6%)	4 (8.5%)	19 (9.9%)	>0.999
	Aspirin	20 (8.4%)	3 (6.4%)	17 (8.9%)	0.772
Admission observations	Systolic blood pressure *(mmHg)*	121 ± 20	120 ± 22	121 ± 19	0.554
	Heart rate *(bpm)*	84 ± 23	89 ± 21	83 ± 23	**0.047**
**Admission investigations**					
Presenting 12-lead ECG *N (%)*	Normal	64 (28%)	14 (33%)	50 (26%)	0.419
	Sinus rhythm	215 (93%)	42 (98%)	173 (92%)	0.317
	ST elevation	62 (27%)	14 (33%)	48 (26%)	0.359
					**0.026**
	LBBB	9 (4.1%)	0 (0%)	9 (5.1%)	
	RBBB	7 (3.2%)	2 (4.8%)	5 (2.8%)	
	IVCD	2 (0.9%)	2 (4.8%)	0 (0%)	
Bloods	Creatinine (mg/dl)	101 ± 83	116 ± 110	98 ± 76	0.212
	eGFR (ml/min/m^2^)	76 ± 23	72 ± 26	77 ± 22	0.505
	Potassium (mmol/L)	4.30 ± 0.59	4.23 ± 0.56	4.32 ± 0.60	0.406
	C-reactive protein (mg/L)	65 ± 86	67 ± 87	65 ± 85	0.346
	Elevated CRP	176 (76%)	28 (62%)	148 (79%)	**0.021**
	WCC *(10^9^/L)*	9.8 ± 4.3	9.9 ± 4.5	9.8 ± 4.3	0.903
	Peak troponin *(g/L)*	11 842 ± 24 209	10 963 ± 23 831	12 046 ± 24 352	0.125
	Elevated troponin	188 (91%)	32 (89%)	156 (91%)	0.750
	Peak troponin *(xULN)*	767 ± 1 570	750 ± 1 589	771 ± 1 570	0.255
	NT-proBNP *(pg/mL)*	1333 ± 5525	1570 ± 4694	1272 ± 5734	0.628
	Elevated NT-proBNP	32 (19%)	5 (14%)	27 (20%)	0.451
Echocardiogram findings	LVEDD *(mm)*	48.1 ± 6.3	46.3 ± 5.9	48.5 ± 6.4	0.073
	LVEDV *(mL)*	110 ± 33	104 ± 23	112 ± 35	0.296
	Left ventricular dysfunction				0.569
	No LVSD	139 (65%)	31 (74%)	108 (63%)	
	Mild LVSD	24 (11%)	4 (9.5%)	20 (12%)	
	Moderate LVSD	28 (13%)	5 (12%)	23 (13%)	
	Severe LVSD	22 (10%)	2 (4.8%)	20 (12%)	
	LVEF *(%)*	51 ± 11	51 ± 10	51 ± 11	0.962
**In-hospital management**					
Place of care *N (%)*	Cardiology ward	162 (68%)	30 (65%)	132 (68%)	0.809
	General medicine ward	57 (24%)	11 (24%)	46 (24%)	
	Other ward	21 (8.8%)	5 (11%)	16 (8.2%)	
Advanced therapies *N (%)*	Inotropes	22 (9.2%)	4 (8.7%)	18 (9.4%)	>0.999
	IVIG	2 (0.8%)	0 (0%)	2 (1.0%)	>0.999
	IV steroids	16 (6.7%)	2 (4.3%)	14 (7.3%)	0.744
	RRT	15 (6.3%)	3 (6.5%)	12 (6.2%)	>0.999
	IABP	1 (1.6%)	1 (11%)	0 (0%)	0.148
	ECMO	2 (3.3%)	2 (22%)	0 (0%)	**0.020**
Discharge medication *N (%)*	Aspirin or NSAID	86 (36%)	14 (30%)	72 (37%)	0.347
	Colchicine	50 (21%)	9 (19%)	41 (21%)	0.763
	RAAS inhibitor	109 (45%)	15 (32%)	94 (48%)	**0.041**
	Beta blocker	108 (45%)	10 (21%)	98 (51%)	**<0.001**
	MRA	12 (5.0%)	1 (2.1%)	11 (5.7%)	0.469
	Diuretic	24 (10.0%)	3 (6.4%)	21 (11%)	0.586
	Statin	44 (18%)	13 (28%)	31 (16%)	0.065
	Amiodarone	4 (1.7%)	1 (2.1%)	3 (1.5%)	0.583
	Immunosuppressant	33 (14%)	5 (11%)	28 (14%)	0.497
**Clinical outcomes**					
Death *N (%)*		13 (5.3%)	3 (6.2%)	10 (5.0%)	0.721

CMR: cardiac magnetic resonance imaging; CKD: chronic kidney disease; CRP: C-reactive protein; ECMO: extracorporeal membrane oxygenation; IABP: intra-aortic balloon pump; IVCD: intraventricular conduction delay; IV: intravenous; IVIG: intravenous immunoglobulin; LBBB: left bundle branch block; LVEDD: left ventricular end diastolic dimension; LVEDV: left ventricular end diastolic volume; LVEF: left ventricular ejection fraction; LVSD: left ventricular systolic dysfunction; MI: myocardial infarction; MRA: mineralocorticoid receptor antagonist; NSAID: non-steroidal anti-inflammatory drug; NT-proBNP: N-terminal pro B-type natriuretic peptide; PAD: peripheral arterial disease; RAAS: renin angiotensin aldosterone system; RBBB: right bundle branch block; RRT: renal replacement therapy; and WCC: white cell count.

On admission, blood tests were broadly similar between cohorts, although more CMR-confirmed AM patients had an elevated C-reactive protein (*P *= 0.021). Echocardiogram findings were similar between the groups, with no differences in ejection fraction or degree of LVSD. Patients received similar in hospital management, although two CS-AM patients received extracorporeal membrane oxygenation (ECMO) compared to no CMR-confirmed patients (*P *= 0.020). On discharge, patients with CS-AM were significantly less likely to receive RAAS inhibitors or beta-blockers than their CMR-confirmed counterparts (*P *= 0.041 and *P *< 0.001 respectively). Other medications, including aspirin, NSAIDs, colchicine and diuretics, were prescribed to a similar proportion of patients in each group.

#### Outcomes

**Figure 5:**
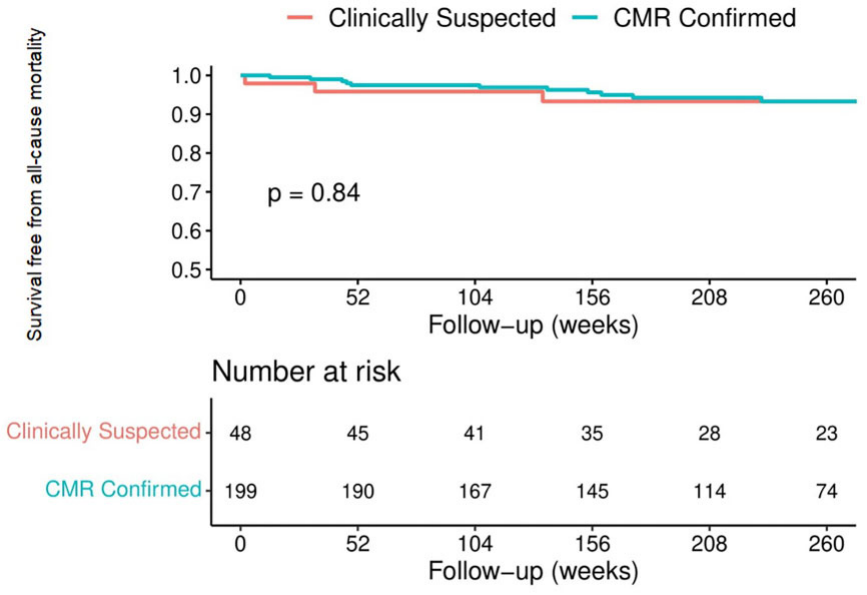
Kaplan–Meier analysis of survival for clinically suspected vs. CMR-confirmed acute myocarditis.

During a median follow-up of 52 months (IQR 32–76) 5.7% (*n* = 14) patients died, with no significant difference between CS-AM and CMR-confirmed AM patients (*P *= 0.721). Thirteen patients died, 6.2% (*n* = 3) in the CS-AM group and 5.0% (*n* = 10) in the CMR-confirmed group (*P *= 0.739). Kaplan–Meier analysis (*Figure 5*) of survival free from death showed no significant difference between the CS-AM and CMR-confirmed cohorts (*P *= 0.84).

### Differences between published AM cohorts with varying diagnostic criteria

We sought to investigate the effect of different diagnostic criteria on reported outcomes in AM studies by reviewing the literature. To this aim, we identified 23 observational studies reporting on cohorts of patients with AM. Of these, 5 included patients identified with ICD codes, 2 included patients with CS-AM, 10 with primarily CMR-confirmed AM and 8 with EMB-confirmed AM (*Table 3*).

**Table 3:** Inclusion criteria and outcomes for published acute myocarditis studies

Reference	Inclusion criteria	N	Outcome
**ICD codes corresponding to AM**			
Ozierański et al., 2021^11^	ICD-10 codes: I40, I40.0, I40.1, I40.8, I40.9, I41, I41.0, I41.1, I41.2, I41.8, I51.4, B33.2 National healthcare insurance database	19 978	In-hospital mortality: 2.5% 5 year mortality (aged 0–20 year): 2% 5 year mortality (aged 80+): 74%
Wong et al., 2020^19^	ICD-10 codes: not detailed Patients undergoing CMR showing MI or sarcoidosis excluded Health board database	178	Mean 4.5 year mortality: 3.4% Mean 4.5 year MACE: 14.6%
Ogunbayo et al., 2017^17^	ICD-9-CM codes: 422.0, 422.90–422.93, 422.99, 429.0 National database	31 220	In-hospital mortality (without heart block): 2.7%
Subahi et al., 2018^16^	ICD-9-CM codes: 422.0, 422.90–422.93, 422.99, 429.0 National database	6642	In-hospital mortality (without AF): 5.4% In-hospital mortality (with AF): 8.8%
Moady et al., 2022^49^	ICD-9 codes: not detailed	59	In-hospital complications (AF, VT, pulmonary congestion): 23.7%
**Clinically suspected AM**			
Gräni et al., 2017^20^	CS-AM with no late gadolinium enhancement on CMR	376	Median 4.7 year follow up Annualised event rates for MACE: 2.1% Annualised event rates for death: 0.9%
Inaba et al., 2017^21^	CS-AM	138	In-hospital mortality: 14.5%
**Primarily CMR confirmed**			
Cannata et al., 2022^2^	CMR-confirmed	199	Median 4.4 year mortality: 5.0% Median 4.4 year MACE: 5.5%
Ammirati et al., 2018^23^	CMR-confirmed or histologically confirmed (EMB/post-mortem/explanted heart; 14%)	443	In-hospital cardiac death/HTx: 3.2% In-hospital death: 2.3% 1 year cardiac death/HTx: 3.0% 5 year cardiac death/HTx: 4.1%
Aquaro et al., 2017^28^	CMR-confirmed (95%) or EMB-confirmed (5%)	374	Median 4.3 year MACE: 7.8% Median 4.3 year sudden cardiac death: 1.1%
Sanguineti et al., 2015^25^	CMR-confirmed	203	Mean 1.6 year MACE: 10.8% Mean 1.6 year mortality: 0%
Bohbot et al., 2022^29^	CMR-confirmed	388	Median 7.5 year MACE: 10.5% Median 7.5 year cardiac death/HTx: 1.5%
Ammirati et al., 2017^24^	CMR-confirmed (80%) or EMB-confirmed (27%)	187	In-hospital mortality: 5.3% In-hospital death/HTx: 7.5%
Imazio et al., 2018^22^	CMR-confirmed	71	Mean 5.1 year MACE: 5.6% Mean 5.1 year mortality: 0%
Gräni et al., 2017^20^	AM with late gadolinium enhancement on CMR	294	Median 4.7 year follow up Annualised event rates for MACE: 4.8% Annualised event rates for death: 1.7%
White et al., 2019^26^	AM undergoing CMR (72% with LGE); not necessarily CMR-confirmed	100	1 year mortality: 0% 1 year MACE: 0%
Younis et al., 2020^27^	AM (73% undergoing CMR of whom 83% had LGE)	322	In-hospital mortality: 0% In-hospital MACE: 7.8% 1 year mortality: 0%
**EMB proven**			
Ammirati et al., 2019^30^	Histologically confirmed (EMB/post-mortem/explanted heart) with LVEF < 50%	220	60-day cardiac death/HTx: 21.4% 7 year cardiac death/HTx: 34.5%
Grün et al., 2012^31^	EMB-confirmed	222	Median 4.7 year cardiac death: 15.0% Median 4.7 year mortality: 19.2%
Anzini et al., 2013^32^	EMB-confirmed	82	Mean 12.3 year mortality: 28.0% Mean 12.3 year cardiac death: 20.7%
Grogan et al., 1995^33^	EMB-confirmed	27	5 year mortality: 44.0%
Mason et al., 1995^34^	EMB-confirmed with LVEF < 45%	111	5 year mortality: 30.6%
McCarthy et al., 2000^35^	EMB-confirmed	147	Mean 5.6 year mortality: 32.7%
Magnani et al., 2006^36^	EMB-confirmed	112	1 year death/HTx: 21.4% 5 year death/HTx: 43.8%
Caforio et al., 2007^37^	EMB-confirmed	174	Median 2 year death/HTx: 14.9%
Greulich et al., 2020^38^	EMB-confirmed viral myocarditis	183	Median 10.1 year mortality: 39.3%

AF: atrial fibrillation; AM: acute myocarditis; CMR: cardiac magnetic resonance imaging; CS: clinically suspected; EMB: endomyocardial biopsy; HTx: heart transplant; ICD-9/ICD-10: International Classification of Diseases 9th/10th Revision; LGE: late gadolinium enhancement; LVEF left ventricular ejection fraction; MACE: major adverse cardiac events; and VT: ventricular tachycardia.

Almost all studies were observational in nature, describing single- or multi-centre data. Studies investigating ICD-identified AM, CS-AM and CMR-confirmed AM were contemporaneous (2015 onwards), compared to some EMB-confirmed AM studies which were from the 1990s and 2000s. Although endpoints varied, almost all studies described mortality and/or a composite measure of major adverse cardiovascular events either whilst in hospital or at long-term follow up.

Although heterogeneous, studies on AM cases identified using ICD codes reported an in-hospital mortality ranging from 2.5 to 8.8%.^16–18^ Longer-term outcomes were less consistently reported, with one study describing a mortality at mean follow up of 4.5 years of 3.4%,^19^ and another study reporting on 5-year survival stratified by sex and age ranging from 98.0% in males <20 years old to 25.6% in male >80 years old.^11^

Only two studies were identified with CS-AM patients who had no further confirmatory testing.^20,21^ In a cohort of CS-AM patients with no late gadolinium enhancement on CMR, Gräni and colleagues reported an annualized event rate of 2.1% for major adverse cardiac events (MACE) and 0.9% for mortality at median follow-up of 4.7 years.^20^ Conversely, a 2017 study by Inaba and colleagues reported a 14.5% in-hospital mortality for CS-AM patients, although 30% of patients presented with fulminant AM requiring advanced life support (percutaneous cardiopulmonary support, ventricular assist devices).^21^

Studies primarily including patients with CMR-confirmed AM also generally had favourable outcomes, with in-hospital mortality ranging from 0 to 5.3%.^20,22–27^ Notably, the study with the highest in-hospital mortality (5.3%) had a mixed cohort, with 27% of cases being EMB-confirmed and the rest CMR-confirmed.^24^ It is unclear whether the EMB-confirmed patients had a disproportionately higher mortality than the CMR-confirmed patients in this study. Longer term follow-up in the CMR-confirmed cohorts again showed overall favourable outcomes across all studies identified, with mortality at 1.0–7.5 years ranging from 0 to 5.0%.^2,22,23,25–29^

Conversely, cohorts with EMB-confirmed AM had generally worse outcomes. Although studies were heterogeneous in terms of clinical outcome definitions and length of follow-up, multiple contemporary and historical studies showed long-term (range 2.0–12.3 years) mortality rates ranging from 14.9 to 44.0%.^30–38^

## Discussion

In this study, we aimed to investigate the complexity and significance of diagnosis in AM. There are 3 main findings. First, using ICD-10 coding to identify patients with AM is inaccurate, and outcomes between ICD-10 code-identified patients with confirmed and refuted AM are different. Second, we found differences in clinical presentation and management between CMR-confirmed AM and CS-AM patients. Third, we identified important differences in clinical outcomes in published cohorts of AM depending on inclusion criteria. These findings highlight the potential challenges in making an accurate diagnosis in AM. They also underline the significant impact the choice of diagnostic criteria can have in influencing prognosis in clinical practice but also research cohorts.

### Validation of ICD-10 diagnostic codes in acute myocarditis

Several studies investigating AM have relied on ICD-10 codes for case identification. Our findings suggest that the accuracy of ICD-10 codes in case identification for AM is poorer than for HF. In our study, relevant ICD-10 codes in the first three coding positions had a PPV of 36.4% for CS-AM (including patients with and without a CMR) and 32.6% for purely CMR-confirmed AM, compared to 94.9% for HF.

In order to exclude suboptimal coding practice at out Centre as a cause of low PPV for AM ICD-10 codes, we compared accuracy of ICD-10 codes with HF and compared this with published studies. Our findings are consistent with the literature: a 2010 review of HF validation studies^13^ reported PPVs of HF ICD-10 codes as the ‘main’ or ‘principal’ diagnosis ranging from 80 to 100%. A 2014 meta-analysis found similar results, with PPVs ≥87% and specificity ≥95% for most studies analysed.^39^ A more recent analysis of 200 patients in France showed that, amongst patients with a HF ICD-10 code as main or secondary code, 88.5% had a definite or potential diagnosis of HF, with the PPV even higher for those with a ‘main’ diagnosis of HF (95.9%).^40^ To our knowledge, only one other study has applied a similar methodology to investigate the accuracy of ICD-10 codes in AM, using the Danish National Patient Registry.^41^ Sundbøll and colleagues reported a PPV of 64% for a gold standard of CS-AM, which despite being somewhat higher than our PPV of 36.4%, was significantly lower than other cardiovascular diseases assessed in their manuscript, including myocardial infarction, stable angina, HF, valvular disease, and arrhythmias.^41^

This discrepancy reflects the diagnostic complexity of AM, particularly compared with diagnostic criteria for HF. Cardiac magnetic resonance and EMB are more resource and labour intensive and therefore less accessible than transthoracic echocardiogram, leading to diagnostic uncertainty and misclassification. Furthermore, the broad spectrum of clinical presentations of AM compared to HF complicates diagnosis, as do disease processes that may mimic AM (such as acute cardiac injury secondary to critical illness).

Further to this, searching our trust EHRs for ICD-10 codes identified 122 patients with CMR-confirmed AM. Using more sophisticated methods of record interrogation, including the CogStack system,^15^ we identified a further 77 patients, suggesting that in addition to poor accuracy, using ICD-10 codes risks missing AM cases. However, it should be noted that the sensitivity will vary according to local coding practices.

A comparison of outcomes in patients with relevant ICD-10 codes showed a significantly higher all-cause mortality amongst patients whose diagnosis was refuted than those with confirmed AM. Acute myocarditis mimics such as acute coronary syndromes or arrhythmogenic cardiomyopathy have different management strategies, and mortality rates for these mimics differ from CMR-confirmed AM, which is associated with an overall favourable prognosis.^2,20,22,25–29^ This finding further underlines the limitation of ICD codes for identifying AM cases, risking significant selection bias and possible overestimation of mortality rates.

Our findings suggest that relying solely on the use of ICD codes for case identification in AM research is insufficient, and research should instead focus on EMB- or CMR-confirmed AM cases. This is particularly relevant in the context of the ongoing coronavirus disease 2019 (COVID-19) pandemic, where AM has been associated with both the virus and vaccines against it.^42–44^ Due to the rapidly evolving nature of the pandemic and urgent need for data, many early studies relied on ICD codes to identify cases of COVID-19- and vaccine-associated AM. This approach, however, may lead to study populations which are not truly representative of the disease processes being investigated. Similarly, the Global Burden of Disease Study 2019 is based on cases of AM identified using ICD codes.^45^ Although it is not feasible to study diseases with complex diagnostic pathways such as AM on a global level using only confirmed cases, this does highlight limitations that must be considered when interpreting the GBD Study's conclusions on AM.

### Comparison of clinically suspected and CMR-confirmed acute myocarditis

In addition to considering discrepancies between EMB- and CMR-confirmed AM, we investigated CS-AM patients. Analysis of patients with CS-AM (but not confirmed by CMR) (*n* = 48) vs. CMR-confirmed AM (*n* = 199) at our Centre highlighted some important differences between the two cohorts. Although demographic data, clinical presentation and comorbidities were similar between groups, patients with CS-AM were less likely to be discharged with RAAS inhibitors and beta-blockers than those with CMR-confirmed AM (despite more patients with CS-AM taking RAAS inhibitors pre-admission). This is despite a lack of difference in EF, rates of LV systolic dysfunction, or hypertension to explain different rates of prognostic anti-remodelling medication prescription. No difference in death was noted over the follow up period between the two cohorts.

Of note, the two patients undergoing ECMO in this study were both in the CS-AM cohort. It is likely that these patients were too unstable to undergo EMB or CMR, and therefore a definitive diagnosis was not made. This highlights a potential selection bias: critically unwell patients and/or patients with early death may be disproportionately excluded from CMR- or EMB-confirmed cohorts due to lack of opportunity for confirmation of AM diagnosis.

There is a lack of high quality, interventional, randomized trial data in the management of AM, and much guidance is dictated by expert consensus.^1^ The ESC position statement recommends managing ventricular dysfunction in line with ESC guidelines on HF, namely anti-remodelling agents. However, in the absence of HF, these medications are currently not indicated. The reasons for increased prescriptions in patients with CMR-confirmed AM are not clear, but might relate to increased confidence in the diagnosis of AM following positive CMR, and awareness of pre-clinical *in vivo* data suggesting that beta-blockers and RAAS inhibitors may be effective in preventing myocardial injury and scar deposition in AM.^46^ Our findings suggest that despite no significant difference in EF or rates of LV dysfunction between the CMR-confirmed and CS-AM cohorts, the former received more anti-remodelling agents.

### Differences between published AM cohorts with varying diagnostic criteria

Our findings suggest that populations diagnosed with AM through EMB and CMR differ in patient characteristics and outcomes. The reasons for this are likely to be multifactorial: Although EMB and CMR are the two most frequent investigations used to confirm a diagnosis of AM, access to these investigations may be limited, with both requiring user/operator expertise, and EMB additionally requiring access to laboratory expertise. However, patients undergoing EMB and CMR may differ for reasons beyond accessibility of the investigations. Despite EMB generally being accepted as a safe procedure with low complication rates,^4^ a large study in the USA reported a total complication rate of approximately 10% following native-heart EMB.^47^ In patients with a mild presentation of suspected AM, undergoing an invasive procedure with a risk of significant complications may not be justified, and non-invasive confirmation with CMR may be more suitable. Conversely, EMB may be more commonly performed in patients where knowing aetiology will change management, such as in fulminant myocarditis, or autoimmune-mediated/inflammatory myocarditis (eosinophilic, lymphocytic, or giant-cell myocarditis). Indeed, in contrast to EMB, CMR is not always able to identify the cause of acute inflammation. Nevertheless, validation studies have shown that the 2018 Lake Louise Criteria have a sensitivity of 95.3% and a specificity of 86.7% with EMB as a reference, supporting acceptable diagnostic performance of CMR.^48^

Our review of the literature supports the notion that clinical outcomes differ according to diagnostic criteria for AM. Classifying published cohorts of AM patients into primarily CMR-confirmed AM vs. EMB-confirmed AM showed that at long-term follow-up, mortality ranged from 14.9 to 44.0% for EMB-confirmed AM compared to only 0–4.1% for CMR-confirmed AM. Patients identified using ICD-10 codes showed in-hospital mortality rates of 2.5–8.8%, although limited data were available on long-term outcomes. We noted significant heterogeneity in outcomes in the two CS-AM studies identified, with Inaba and colleagues reporting an in-hospital mortality rate of 14.5%, with 30% of these patients presenting with fulminant myocarditis. This high mortality compared to the study by Gräni and colleagues suggests that the CS-AM cohort in itself is heterogeneous, likely due to the lack of specific diagnostic criteria. Taken together, these findings illustrate the risk of selection bias when using different inclusion or exclusion criteria and emphasise the importance of consistent and standardized diagnostic criteria for AM to ensure reliable comparisons between studies.

CS-AM is described by the ESC working group as an aid to the selection of patients requiring further diagnostic investigations.^1^ Our analysis, which highlights differences in CS-AM patients compared to both CMR- and EMB-confirmed AM patients, supports this. We suggest that patients with CS-AM should undergo either CMR or EMB for confirmation, both in clinical practice and in research.

### Limitations

This study was a retrospective cohort study of prospectively collected data at a single hospital Trust in London, UK. Our patient population may not be entirely reflective of the national or international populations, limiting the generalizability of this study. Although EHRs were thoroughly screened, cases of AM may have been missed. Furthermore, cardiac endpoints were captured at our institution only, and death elsewhere may have been missed. Furthermore, due to the lack of routine EMB and post-mortem studies we were unable to account for the aetiology of AM. Additionally, given the observational study design, decisions on management and investigation of patients were taken by the lead clinician responsible for the patient, introducing the possibility of bias. Nevertheless, clinical presentations and outcomes in our cohort are comparable to previously published multi-centre data.^23^

Additionally, of the 48 patients with CS-AM at our Centre, 41 did not undergo CMR with the remaining seven undergoing CMR but not meeting Lake Louise criteria. It is possible that these two cohorts may have had different clinically characteristics, although the numbers in this study are too small to investigate this further. Furthermore, there is no clear consensus as to whether CS-AM is defined as patients not undergoing CMR, patients with a negative CMR, or a combination of both.

Furthermore, the nature of our study design meant that chronic forms of myocardial inflammation could not be excluded. However, we excluded patients with elective, non-acute admissions, which may have reduced the risk of chronic myocardial inflammation patients being included. Furthermore, numbers of patients with previous myocarditis were low and did not differ between CS-AM and CMR-confirmed AM groups. Furthermore, given that patients included did meet the relevant diagnostic criteria for AM, this limitation is likely to be inherent to studies in the literature as well, and further highlights limitations in current diagnostic criteria.

Finally, in our review of published AM cohorts, we grouped studies with primarily CMR-confirmed AM together. Although the majority of studies described cohorts where ≥80% of patients had CMR-confirmed AM,^2,22–25,28,29^ three other studies had more heterogeneity in inclusion criteria.^20,26,27^ However, as there were insufficient studies to break down inclusion criteria further, we consider studies as primarily describing CMR-confirmed if >50% of patients underwent CMR diagnostic for AM.

## Conclusion

Diagnosis of AM is complex, due to its heterogeneous clinical presentations, many clinical mimics, and the limitations of diagnostic tests. Nevertheless, accurate diagnosis is important from both a clinical and academic perspective. We show that ICD-10 codes, often used in AM research, lack sensitivity and specificity in case identification compared to their performance in HF, and that patients with refuted AM have a higher mortality than those with confirmed AM. Furthermore, we have highlighted important differences in management and outcomes of patients depending on the criteria used to diagnose AM. This study has highlighted the potential selection biases when interpreting cohorts of AM patients, and we suggest the standardization of inclusion criteria for AM studies to allow robust results and valid comparisons between studies.

## Notes

**Conflicts of interest:** M.G. is a member of the Scientific Advisory Boards of Trizell Holding SA, Lausanne and DINAQR AG, Zurich-London and is founder, consultant and equity holder in Purespring Therapeutics and Forcefield Therapeutics, London, which operate in the field of gene therapy and cardioprotection. A.M.S. reports an advisory board association with Forcefield Therapeutics, London. P.S. reports an advisory board association with Abbott Medical. S.P. reports an advisory board association with Bayer and Pharmacosmos.

## Data availability

The aggregate data underlying this article can be shared on reasonable request to the corresponding author.
